# Effect of Cold Shock Pretreatment Combined with Perforation-Mediated Passive Modified Atmosphere Packaging on Storage Quality of Cucumbers

**DOI:** 10.3390/foods11091267

**Published:** 2022-04-27

**Authors:** Fucheng Wang, Si Mi, Bimal Chitrakar, Jinsong Li, Xianghong Wang

**Affiliations:** 1 College of Food Science and Technology, Hebei Agricultural University, Baoding 071001, China; laster2022@163.com (F.W.); misi@hebau.edu.cn (S.M.); bimal@hebau.edu.cn (B.C.); 2 Bureau of Agriculture and Rural Areas of Qinhuangdao, Qinhuangdao 066000, China; april202204@163.com

**Keywords:** cold shock, MAP, cucumber, storage, VOCs

## Abstract

This study evaluated the application of cold shock combined with perforation-mediated passive modified atmosphere packaging technology (CS-PMAP) for cucumber preservation through physicochemical, sensory, and nutritional qualities. The effectiveness of CS-PMAP in maintaining the quality of fresh cucumbers was studied; cucumbers were pretreated with cold shock and then packed into perforated polyethylene bags (bag size of 20 × 30 cm; film thickness of 0.07 mm; and two holes in each bag with a diameter of 6 mm), while the cucumbers without cold shock were considered as the control. Storage of the samples was performed at (13 ± 2) °C for 20 days to determine the quality changes in terms of gas composition, weight loss, skin color, texture, total soluble solids (TSS), ascorbic acid, malondialdehyde (MDA), and volatile organic compounds (VOCs). The CS-PMAP showed a significant improvement in maintaining firmness, TSS, ascorbic acid, and flavor profile of cucumbers; the control samples without cold shock showed higher weight loss and MDA levels. Results of this study confirmed that CS-PMAP has potential use in the storage of cucumbers.

## 1. Introduction

Cucumbers (*Cucumis sativus* L.) are cylindrical fruits, normally considered as vegetables because of the way they are used in food preparation; they are widely cultivated in China [1]. They are rich in minerals, vitamins, and flavonoids but also high in moisture, which makes them susceptible to deterioration after harvest due to water loss, yellowing color, and microbial action; the shelf life of fresh cucumbers is limited to 3–5 days at room temperature [2]. To maintain the post-harvest quality of cucumbers, several treatment methods have been studied, including chemical preservatives [3], physical treatments [4,5], coatings [2,6,7], modified atmosphere packaging [8], and co-preservations [9,10]. However, with a rising demand of safe foods from consumers, a safer and more effective method of preservation to replace the traditional preservation methods for cucumber is very important.

The pre-cooling process is crucial in the cold supply chain management of vegetables and fruits. In recent years, cold shock treatment (CS) has received extensive attention as a modified pre-cooling method. It is a physical preservation method of fruits and vegetables, which is usually performed with ice water [11]. Simple, convenient, safe, and low cost are the characteristics of CS treatment. Therefore, it is suitable for high-volume processing in both small and large scales industries, especially in developing countries. Several studies have shown that cold shock is able to maintain the quality of sweet cherry [11]; reduce lignification of asparagus [10]; inhibit the decrease of firmness in banana [12]; and extend the shelf life of avocado fruits [13].

Modified atmosphere packaging (MAP) is a method to slow down food deterioration by using different kinds of packaging films and different concentrations of oxygen (O_2_) and carbon dioxide (CO_2_). MAP can be done in two ways: The first one is active MAP, where a product-specific gas or gas mixture is used in the pack, while the second one is passive MAP, where a spontaneous change in the gas composition occurs from the fresh food that respires in the packaged MAP [14] and no additional gas supply is practiced. Therefore, passive MAP is more cost-effective than active MAP and is reported to be applicable to a wide range of fresh fruits and vegetables [15]. Furthermore, for passive MAP, the choice of packaging films is more important. Agricultural products consume O_2_ through respiration and produce CO_2_ and water vapor during storage [16]. Most films commonly used in MAP have limitations in permeation properties, including high barrier properties to water vapor and unbalanced gas transmission rate, resulting in a favorable condition for anaerobic bacteria and the development of strange odors, among others. It has been reported that O_2_ and CO_2_ levels obtained with conventional MAP are rarely sufficient to maintain high respiratory product quality throughout storage [17]. Perforations with suitable numbers and dimensions have been discovered to promote the packaging film permeability and quickly achieve an O_2_ and CO_2_ balance inside the package [18].

The present work was aimed to evaluate the application of perforation-mediated passive modified atmosphere packaging (PM-PMAP) treatment, combined with CS for maintaining the post-harvest quality of cucumber by assessing the physicochemical, sensory, and nutritional properties of cucumber.

## 2. Materials and Methods

### 2.1. Cucumber Pretreatments and Packaging

Cucumbers were harvested from a vegetable farm located in Changli County, Qinhuangdao City, Hebei Province, China (36°57′ N, 119°45′ E). The picked cucumbers were immediately transported to the laboratory and were sorted based on maturity (color, gloss, and shape) [19], soundness (mechanical injury and insect infestation), and size. Polyethylene (PE) film is colorless, odorless, non-toxic, tasteless, high in mechanical strength, and has moisture resistance in humid environments. Studies have shown that PE has a good preservation effect on fresh fruits and vegetables such as cucumbers, persimmons, and mushrooms [15], thus making it the first choice as packaging material for these products. The sorted cucumbers were randomly divided into four groups, each group containing 45 cucumbers. The first group was immersed in 0 °C ice water for 20 min (CS20). Similarly, the second and third groups were soaked in ice water at 0 °C for 40 min (CS40) and 60 min (CS60), respectively, while the fourth group was not pretreated with ice water (NCS). Then, all groups of cucumbers were put into perforated PE film bags (bag size was 20 × 30 cm; film thickness was 0.07 mm; each bag had two holes having a diameter of 6 mm) and then heat-sealed. Groups were defined as CS20+P, CS40+P, CS60+P, and NCS+P (control group). Finally, all the samples were stored at 13 ± 2 °C for 20 days in a cold storage room; the reason behind choosing that temperature was that cucumbers are susceptible to chill injury at 10 °C or lower temperatures, while they turn yellow at 15 °C or higher [8,20]. Samplings were done on the 0, 4, 8, 12, 16, and 20 days to analyze the following quality attributes: internal atmosphere composition, weight loss, instrumental color, instrumental texture, total soluble solids, ascorbic acid content, malondialdehyde (MDA) content, and volatile organic compounds (VOCs).

### 2.2. Internal Atmosphere Composition

O_2_ and CO_2_ concentrations inside the packages were measured using a portable gas analyzer (OXYBABY^®^ 6.0, Witt, Witten, Germany). The instrument measured O_2_ and CO_2_ concentrations in the headspace through electrochemical and infrared sensors. Results were expressed as percentage (%) of O_2_ and CO_2_ inside the bags. Triplicate measurements were done for each group.

### 2.3. Weight Loss

An electronic scale with an accuracy of 0.1 g was used to weigh the sample at each sampling time, and the weight loss (%) was calculated based on the initial weight. All measurements were done in triplicate.

### 2.4. Skin Color

The color of cucumbers was measured with a chromameter (CR-400, Konica Minolta, Tokyo, Japan). The parameters L* (lightness), a* (red and green chromaticity), and b* (blue and yellow chromaticity) values were used to describe the colors. The instrument was calibrated using a standard white reflector before measurement. For each experimental group, five cucumbers were randomly selected, and triplicate measurements were made for each sample.

### 2.5. Instrumental Texture

Texture was determined according to Yang et al. [21] with slight modifications. A texture analyzer (TMS-Pro, Food Technology Corporation, Sterling, VA, USA) was used to conduct texture profile analysis using 1 cubic cm cucumber samples. A cylindrical probe with a diameter of 35 mm was used at the probe height of 20 mm. A test speed of 1 mm/s was used throughout (before, during, and after) the test with a depression distance of 50% of the compression degree, while the contact force used was 0.5 N. Firmness was expressed as maximum compression force in Newtons (N) and five measurements were taken for each experimental group.

### 2.6. Total Soluble Solids

Cucumbers were mashed into a fine paste using a lab blender. The mash was then squeezed through four layers of cheesecloth, and the juice was used for the TSS assay using a portable digital refractometer (RSD200, AS ONE, Osaka, Japan). Measurements were made on five fruits per experimental group.

### 2.7. Ascorbic Acid

Ascorbic acid content was determined according to Yang et al. [22] with slight modifications. Accurately weighed (20 g) cucumber was put into a crusher to mash into a homogenate with 20 g of the oxalic acid solution The homogenized sample was made to 100 mL with oxalic acid solution in a volumetric flask. The filtered aliquot (10 mL) was titrated with standard 2,6-dichloroindophenol solution until pink color that last for 15 s. The whole process was carried out under dark conditions. The content of ascorbic acid in cucumber samples was calculated as follows.

(1) $$X\;\left(\mathrm{mg}\;\mathrm{per}\;100\;g\right)=\frac{\left(V-V_{0}\right)\times T\times A}{m}\times 100$$

where *X* is the content of ascorbic acid in milligrams per hundred grams (mg/100 g); *V* is the volume of 2,6-dichloroindophenol solution consumed for titration (mL); *V*_0_ is the volume of 2,6-dichloroindophenol solution consumed for titration blank (mL); *T* is milligrams of ascorbic acid per milliliter of 2,6-dichloroindophenol solution (mg/mL); *A* is dilution factor; and *m* is the mass of the sample (g).

### 2.8. Malondialdehyde

Malondialdehyde (MDA) content was measured using the method described by Wang et al. [23] with slight modifications. MDA content was determined using a microplate spectrophotometer (Multiskan Spectrum, Thermo Scientific, Waltham, MA, USA). Cucumber sample (2 g) was ground with 5 mL of trichloroacetic acid (TCA; 10%, *w/v*). After centrifugation at 8000 r/min for 10 min, 2 mL supernatant (2 mL TCA for control) was mixed with 2 mL of thiobarbituric acid (TBA; 0.67%, *w/v*). The mixture was put in a boiling water bath for 20 min. The sample was cooled rapidly and again centrifuged at 8000 r/min for 3 min. The supernatant was taken to determine the optical density (OD) at 532 nm, 600 nm, and 450 nm. Five replicates were performed for each treatment. The MDA content was calculated according to following formula:

(2) MDA content μmolgmF=6.45×OD532−OD600−0.56×OD450×V1×V2V3×m×1000

where 6.45 is the absorbance correction coefficient at wavelength 532 nm; 0.56 is the absorbance correction coefficient at wavelength 450 nm; OD_532_, OD_600_, and OD_450_ are the absorbance values at 532, 600, and 450 nm, respectively; *V*_1_ is the total volume of sample extract (mL); *V*_2_ is the total volume of reaction solution (mL); *V*_3_ is the volume of sample extracts taken (mL); and *m* is the mass of the sample taken (g).

### 2.9. Volatile Organic Compounds

The volatile organic compounds were analyzed following the methods of Li et al. [24], and Song et al. [25] with some modification using gas chromatography–ion mobility spectrometry (GC–IMS) (FlavourSpec^®^, Gesellschaft für Analytische Sensorsysteme mbH, Dortmund, Germany), equipped with an autosampler unit (CTC Analytics AG, Zwingen, Switzerland) that can directly sample from the headspace by using a 1 mL air-tight heated syringe.

Cucumber samples (2.5 g) were cut into small pieces and placed into 20 mL headspace glass sampling vials. Subsequently, samples were incubated at 40 °C for 10 min with an oscillator speed of 500 r/min. After incubation, 1 mL of headspace gas was automatically injected through a syringe at 85 °C. Then, the samples were driven into a GC column by nitrogen at a programmed flow as follows: 2 mL/min for 2 min; linearly increasing to 15 mL/min from 2 to 10 min; linearly increasing to 100 mL/min from 10 to 25 min; held for 5 min. The column type was MTX-5 (15 m × 0.53 mm, 1 μm). The syringe was automatically flushed with a stream of nitrogen before and after each analysis to avoid cross-contamination.

The retention index (RI) of each compound was calculated using n-ketones C4–C9 as external references. Identification of volatile compounds was based on comparison of RI and drift times (Dts) with GC–IMS libraries.

### 2.10. Statistical Analysis

Microsoft Office Excel 2016 was used to perform the basic processing of the obtained experimental data; other statistical analyses were performed using Addinsoft (2021) (XLSTAT statistical and data analysis solution, New York, NY, USA). A basic descriptive statistical analysis was followed by an analysis of variance test (ANOVA) for mean comparisons; 95% confidence intervals (*p* < 0.05) were set throughout the data analysis to identify significant differences. Principal component analysis and partial least squares discrimination analysis were also performed. VOCs were collected and analyzed using the VOCal analysis software accompanying the GC–IMS instrument. The differences in fingerprint profiles were compared by the reporter and galerie plug-ins equipped with the GC–IMS instrument. The VOCs were qualitatively analyzed by applying the NIST and IMS databases built into the Library Search software.

## 3. Results and Discussion

### 3.1. Internal Atmosphere Composition

**Figure 1:**
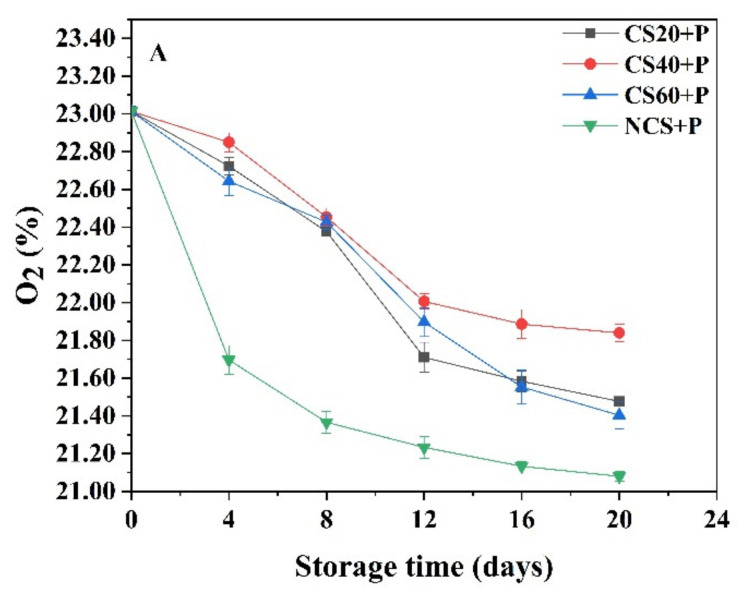

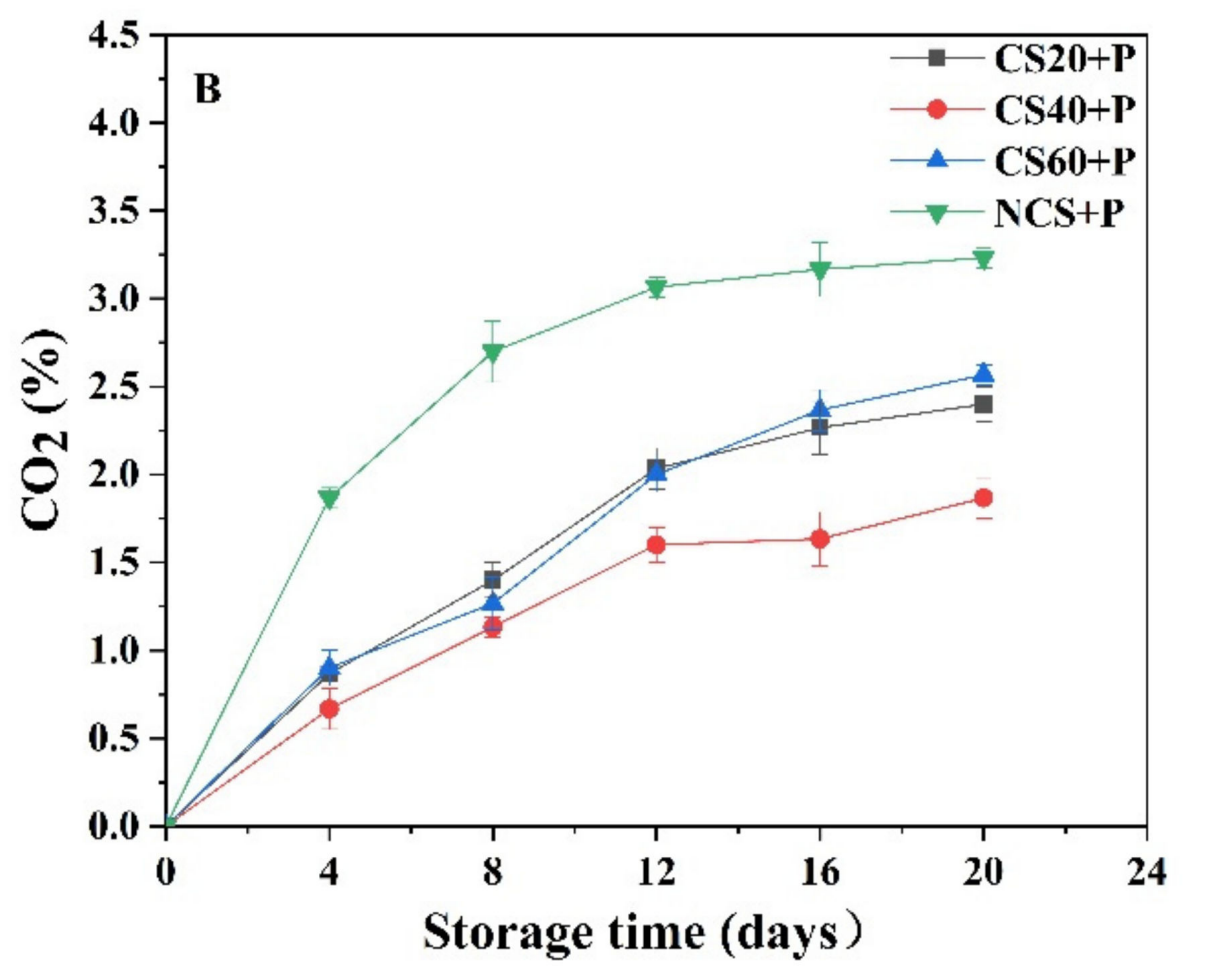
Changes in O_2_ (**A**) and CO_2_ (**B**) content during storage at 13 ± 2 °C in different treatments. CS20+P−packaged after 20 min of cold shock; CS40+P−packaged after 40 min of cold shock; CS60+P−packaged after 60 min of cold shock; NCS+P−packaged after 0 min of cold shock.

It is well known that the changes in gas composition in a MAP system are mainly dependent on storage temperature, film permeability, and product respiration rate [26]. As expected, a decrease in O_2_ concentration as well as an increase in CO_2_ concentration were observed during the storage period at 13 °C (Figure 1A,B). All the treatment groups showed a rapid decrease in O_2_ concentrations during days 4 to 12 (*p* < 0.05); the decrease was slowed down during the subsequent storage period. On day 20, the O_2_ concentrations in the CS20+P, CS40+P, CS60+P, and NCS+P groups decreased from 23.01% to 21.84%, 21.48%, 21.40%, and 21.37%, respectively. The O_2_ in the CS40+P group declined the slowest. Moreover, there was a significant difference between the NCS+P group and the CS40+P group (*p* < 0.05) from the fourth day onwards. When cucumbers were treated by cold shock, the temperature of cucumbers lowered rapidly. The property and structure of the protease related to respiration may be influenced by the cold shock, which can reduce the respiration, so the O_2_ concentration decreased slowly. All the treatment groups showed an increase in CO_2_ levels during the storage period; CS20+P, CS40+P CS60+P, and NCS+P groups exhibited an increase from 0 to 2.40%, 1.87%, 2.57%, and 3.23%, respectively, on day 20. The CO_2_ concentration in the CS40+P group increased the slowest. In addition, the CO_2_ concentration in the CS40+P group was significantly lower than the other two CS treatment groups from the fourth day. It may be that the treatment time of CS20+P was too short, resulting in insufficient reduction of enzyme activities related to respiration, and that the treatment time of CS60+P was too long, causing some irreversible damage to cucumber tissue. In summary, the experimental results indicated that CS regulated effectively the concentrations of O_2_ and CO_2_ in the packaging bags. Similar results were observed in avocado fruits subjected to cold shock with 0 °C ice-water mixture [13]. The CS40+P treatment best inhibited the respiration rate of cucumber fruits, which was beneficial to cucumber storage.

### 3.2. Weight Loss

Cucumbers have a high moisture content (about 95%) [8]. One of the main problems during post-harvest storage of cucumbers is rapid water loss from cucumbers, resulting in weight loss. This may be because cucumbers are only protected by a thin skin structure, and the weight loss of fresh produce in the postharvest process is caused by two factors, namely transpiration and respiration [27]. Transpiration can cause water loss, while respiration can cause dry matter loss [28]. Harvested produce releases water vapor into the surrounding atmosphere through transpiration, while the respiration process uses reserves of organic materials and also releases water vapor [29] to reduce the weight of fruits and vegetables.

Weight loss of cucumbers increased consistently with the prolongation of storage time at 13 °C (Table 1). Started from day 12, weight loss of the NCS+P group was significantly (*p* < 0.05) higher than the other groups. In general, CS-treated samples showed less weight loss compared to NCS-treated samples, and the lowest weight loss was observed in the CS40+P group. This results revealed that CS-treated cucumbers could retain their weight, compared to those with NCS, and the CS40+P treatment was the best combination to prevent cucumber weight loss. The above results showed that cold shock may inhibit the respiration and transpiration of cucumber. This was in agreement with the previous studies on asparagus spears, where the reductions in fresh weight loss by CS were significant during the storage period [10].

### 3.3. Skin Color

**Figure 2:**
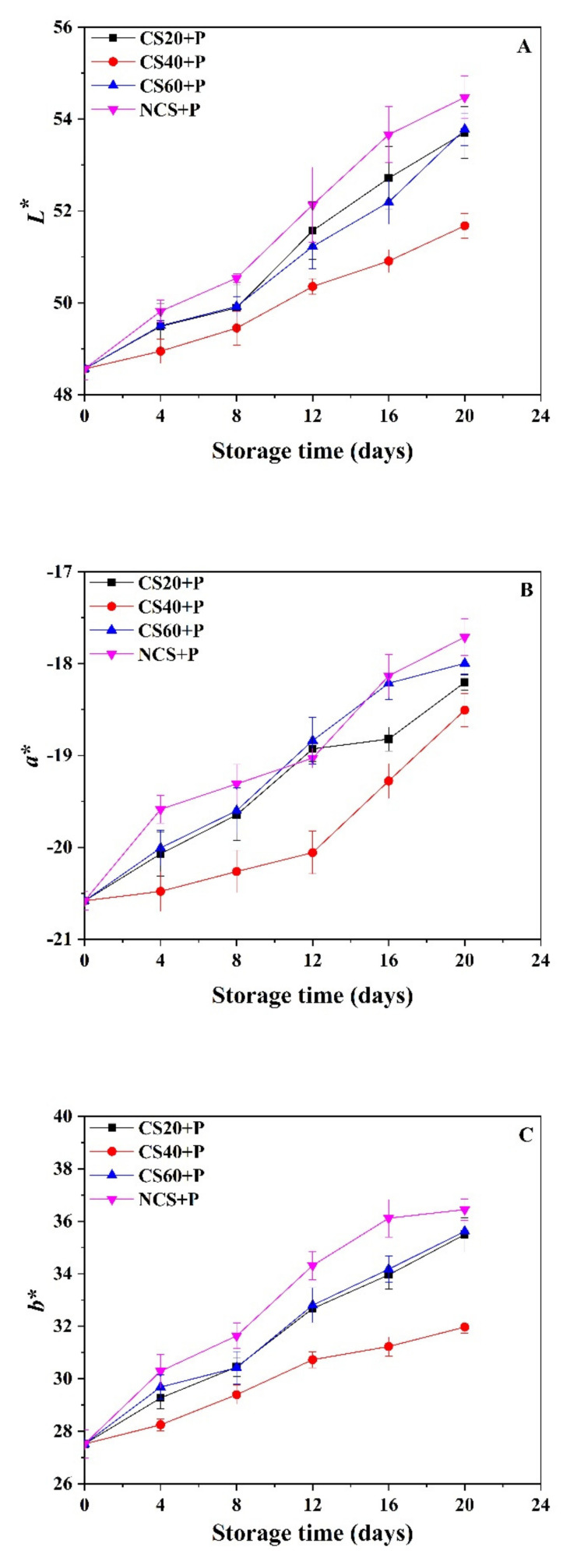
Effect of different treatments on the L* (**A**), a* (**B**), and b* (**C**) values of cucumbers during storage at 13 ± 2 °C. CS20+P−packaged after 20 min of cold shock; CS40+P−packaged after 40 min of cold shock; CS60+P−packaged after 60 min of cold shock; NCS+P−packaged after 0 min of cold shock.

Color is one of the most important quality parameters of consumer acceptance [30], and color changes are important indicators of shelf-life and maturity of cucumbers. The L* values of all treated cucumbers increased with storage period, which was in agreement with a previous study [8]. The L* value of the cucumbers in the NCS+P group remained higher than that of the other groups, and after the 8th day of storage, the L* value of the cucumbers in the NCS+P group was significantly (*p* < 0.05) higher than that of the CS-treated groups. Among the cold shock groups, the L* value of cucumbers in the CS40+P group had the smallest change, namely from 48.56 to 51.68. The results from Figure 2A show that the brightness of cucumbers increased with the extension of the storage period, which may be because of the effect of light, oxidation, and respiration that caused the cucumber to change from its natural green color to white [8]. The cucumbers in the CS40+P group had the smallest change; it may be that CS40+P treatment inhibited the respiration and oxidation of cucumber.

During the storage period, the a* values of CS20+P, CS40+P, CS60+P, and NCS+P groups increased from −20.58 to −18.20, −18.51, and −17.99, respectively. The a* values of these three CS groups were significantly (*p* < 0.05) lower than those of the NCS group on day 4 and day 8, and the a* value of the CS40+P group was consistently maintained significantly (*p* < 0.05) lower than that of other groups. In other words, the greenness of cucumbers in all groups was getting lighter, and the CS40+P group showed the least change. The green color of cucumber is mainly attributed to the presence of chlorophylls, and the decrease in greenness during storage may be caused by the decomposition of chlorophylls. The mechanisms of cold shock treatment are partially based on a decrease in chlorophyllase activity that results in delaying the degradation of chlorophylls [31].

The b* values of all groups increased significantly (*p* < 0.05) with time during the storage period. The mean values of b* for CS20+P, CS40+P, CS60+P, and NCS+P groups increased from 27.52 to 35.50, 31.97, 35.62, and 36.44, respectively, indicating that the color of the cucumber was changing towards a yellow color. The yellow color of cucumber may be caused by the accumulation of carotenoids and flavonoids in the rind [32]. The b* values of the CS-treated group were significantly (*p* < 0.05) lower than those of the NCS-treated group from the eighth day onwards. Moreover, the b* values of the CS40+P group were significantly (*p* < 0.05) lower than those of the CS20+P, CS60+P, and NCS+P groups from day 4 onwards.

The color (L*, a*, and b* values) of cucumbers in CS20+P, CS60+P, and NCS+P groups changed faster than that of the CS40+P group. It may be that the CS20+P treatment time was too short to improve the stress resistance mechanism of cucumber; the CS60+P treatment time was too long, which accelerated the aging of cucumber; and CS40+P treatment improved the stress resistance of cucumber and delayed the color change. In summary, CS40+P treatment had minimal color change; thus, it had a positive effect on the color protection of postharvest cucumbers.

### 3.4. Instrumental Texture

**Figure 3:**
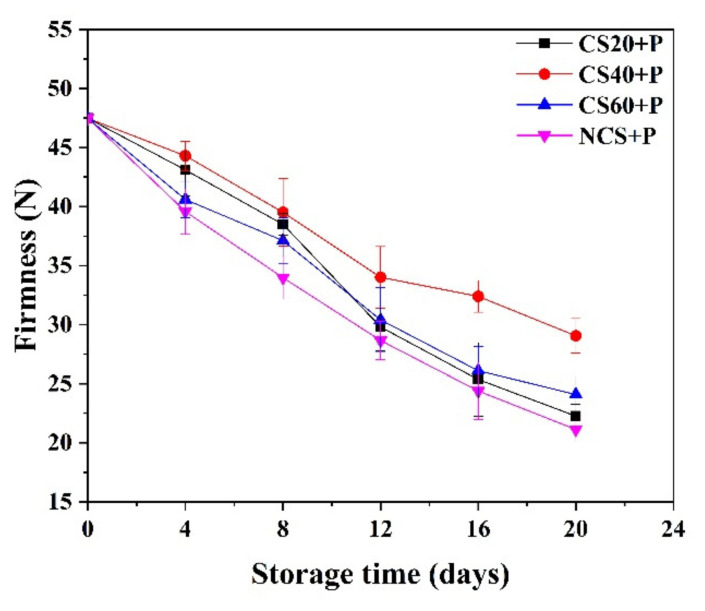
Effect of different treatments on the firmness of cucumbers during storage at 13 ± 2 °C. CS20+P—packaged after 20 min of cold shock; CS40+P—packaged after 40 min of cold shock; CS60+P—packaged after 60 min of cold shock; NCS+P—packaged after 0 min of cold shock.

Firmness is a very important indicator of the quality and shelf-life of agricultural products, as it reflects the biochemical changes that take place in the cell structure [33]. Cucumbers showed a rapid loss of firmness during storage, which was mainly due to water loss [34]. The firmness of cucumbers in all groups decreased gradually during the storage period (Figure 3). Previous research has shown that the hardness of cucumber decreased with storage time [35] as found in our experimental results. At the end of storage, the hardness of the NCS+P group decreased by 55.2% (from 47.48 N to 21.12 N) and was significantly (*p* < 0.05) lower than that of the CS groups. The CS40+P treatment maintained high hardness values of cucumbers throughout the storage period. It may be that the cold shock time in the CS20+P group was not enough to improve the resistance of cucumbers and reduce water loss, and that the cold shock time in the CS60+P group was too long, which made the cucumbers suffer from cold damage and accelerated softening. CS and MAP have been reported to show beneficial effects in preserving texture [36,37], possibly by attenuating respiration and transpiration of cucumber, thereby reducing firmness loss.

### 3.5. Total Soluble Solids (TSS)

**Figure 4:**
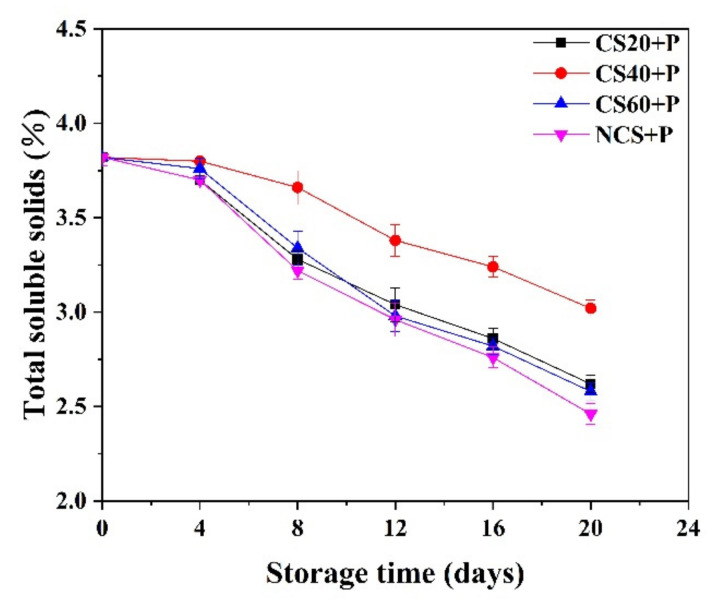
Effect of different treatments on the total soluble solids content of cucumbers during storage at 13 ± 2 °C. CS20+P−packaged after 20 min of cold shock; CS40+P−packaged after 40 min of cold shock; CS60+P−packaged after 60 min of cold shock; NCS+P−packaged after 0 min of cold shock.

The TSS mainly include sugars and acids; TSS is an important parameter to identify fruit maturity and quality [3]. The senescence of fruits is generally involved in a decrease in TSS. The post-harvest deformation of fresh cucumbers (wilting) is caused by changes in water and polysaccharide content that degrade the cell wall [38]. The TSS of all groups gradually reduced with storage time (Figure 4). The decrease of TSS indicated that the cucumber was aging. TSS participated in carbohydrate metabolism of cells [39], thus, causing a reduction of TSS. At end of the 20-day storage period, the TSS value of the CS40+P treatment group was 3.02, which was significantly (*p* < 0.05) higher than that of the other treatment groups. This may be due to the physical effects of CS40+P treatment, which may have inhibited the activity of enzymes associated with carbohydrate metabolism as well as cucumber respiration, thereby maintaining a high TSS level, and the PM-PMAP reduced the gas exchange between the samples and the atmosphere to lower the metabolic rate and slow the hydrolysis of carbohydrates [40].

### 3.6. Ascorbic Acid

**Figure 5:**
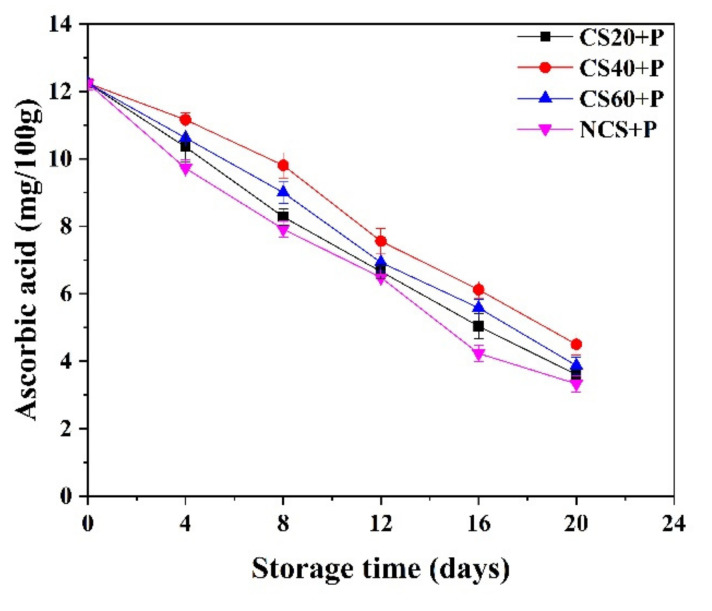
Effect of different treatments on the ascorbic acid content of cucumbers during storage at 13 ± 2 °C. CS20+P−packaged after 20 min of cold shock; CS40+P−packaged after 40 min of cold shock; CS60+P−packaged after 60 min of cold shock; NCS+P−packaged after 0 min of cold shock.

Ascorbic acid is a key nutritional indicator in fruits and vegetables [41] and is an important ingredient to resist oxidant reactions. As can be seen from Figure 5, the ascorbic acid of all treatment groups decreased with time and was significantly (*p* < 0.05) lower than that of fresh cucumber at day 20. Cucumbers in the NCS+P treatment group showed the greatest decline (from 12.24 to 3.33 mg/100g), and the CS40+P treatment group showed the least decrease (from 12.24 to 4.50 mg/100g). From day 4, the CS40+P group was significantly (*p* < 0.05) higher than other three groups. The reason may be that perforated packaging bags formed a good gas balance between cucumber and the outside world, restricted gas exchange, and reduced the oxidation of ascorbic acid. The beneficial effect of polyethylene-based MAP in retaining ascorbic acid in cucumber, green chilies, and jujube was acknowledged by various studies [9,42,43]. In addition, the combination of cold shock treatment and PM-PMAP reduced the respiration rate of cucumber to reduce the consumption of ascorbic acid, which can be reduced by reducing the respiratory rate of the sample [44].

### 3.7. Malondialdehyde (MDA)

**Figure 6:**
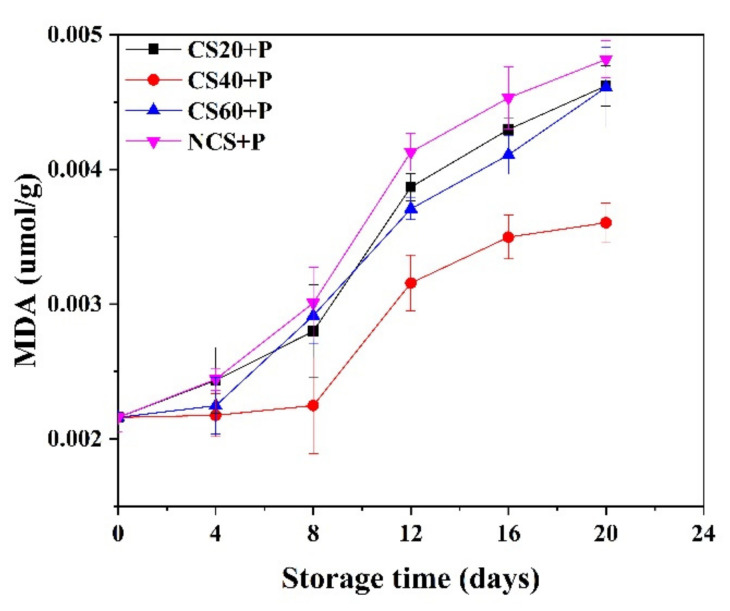
Effect of different treatments on the MDA content of cucumbers during storage at 13 ± 2 °C. CS20+P−packaged after 20 min of cold shock; CS40+P−packaged after 40 min of cold shock; CS60+P−packaged after 60 min of cold shock; NCS+P−packaged after 0 min of cold shock.

As the cucumber fruit matures, the membrane lipid function weakens due to membrane lipid peroxidation. MDA reflects lipid peroxidation related to the permeability and integrity of the membrane [45]. MDA is an indicator of membrane damage [46], and its content can reflect the stress tolerance of plants [47]. Figure 6 shows that the amount of MDA in all treatment groups increased during the storage period. At the beginning of storage, the MDA content in cucumber samples was 0.0022 μmol/g, while at the end of storage, the MDA contents of CS20+P, CS40+P, CS60+P, and NCS+P groups increased to 0.0046, 0.0036, 0.0046, and 0.0048 μmol/g, respectively. The largest increase in MDA was in the control group. From day 8, the MDA content of the CS40+P group was significantly (*p* < 0.05) lower than the other three groups. It was shown that CS40+P treatment had a positive effect on maintaining the antioxidant capacity of plant lipids. It may be that CS20+P treatment time was too short to improve the antioxidant capacity of cucumbers, and that CS60+P treatment time was too long, causing irreversible damage to cucumber tissue and accelerating cucumber oxidation. The study on sweet cherry showed that cold shock treatment inhibited MDA accumulation [11], which was consistent with our findings. This may be because CS treatment reduced the enzymatic activity and water mobility to reduce the accumulation of MDA.

### 3.8. Volatile Organic Compounds

**Figure 7:**
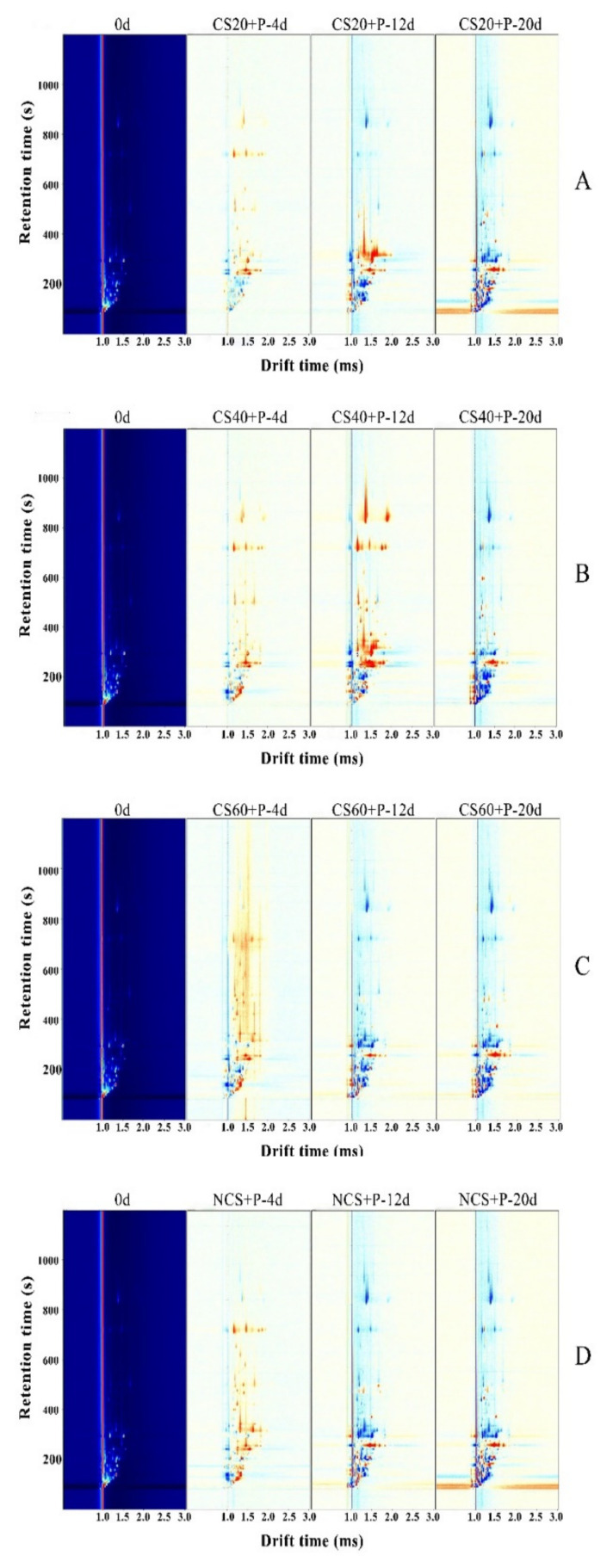
Comparison of the two-dimensional difference spectra of volatile organic compounds GC–IMS in cucumbers under different treatments (**A**): CS20+P; (**B**): CS40+P; (**C**): CS60+P; (**D**): NCS+P.CS20+P−packaged after 20 min of cold shock; CS40+P−packaged after 40 min of cold shock; CS60+P−packaged after 60 min of cold shock; NCS+P−packaged after 0 min of cold shock.

The separation and identification of VOCs in cucumber samples were carried out by the GC–IMS technique. Figure 7 shows two-dimensional difference contrast spectra formed by using the spectrum of cucumber at 0 day as the reference and other spectra deducted from the reference. The horizontal coordinates indicate the ion migration time (drift time, Dt), and the vertical coordinates indicate the retention time (Rt) of gas chromatography. The migration time of VOCs in the samples ranged from 0 to 1.6 ms, and the retention time was concentrated between 100 and 900 s. All VOCs were detected within 30 min. The vertical line at the horizontal coordinate 1.0 in the figure indicates the reactive ion peak (RIP); each dot on both sides of the RIP represent a VOC. In order to distinguish different volatile profiles of CS20+P, CS40+P, CS60+P, and NCS+P, the reactive peak was normalized. In the subtracted spectra, the same concentration of the substance is offset as white; red color indicates that the concentration of the substance is higher than the reference value; the blue color indicates that the concentration of the substance is lower than the reference value; and the darker color indicates a greater difference [48]. Some organic substances appeared at different migration times and formed two or even more signal peaks in the spectrum, due to the higher concentration of the substance; two or more molecules shared a proton or electron, forming a dimer or even a multimer. It can be seen from the figure that the information of gas-phase ion mobility spectra of cucumber samples under different treatment conditions showed some differences during storage, indicating that the content of volatile organic substances in cucumber changed during storage.

In Table 2, a total of 53 volatile components (monomers and dimers of some substances) could be unambiguously characterized, including 13 alcohols, 18 aldehydes, 6 ketones, 5 esters, 4 acids, 2 ethers, and 5 others.

**Figure 8:**
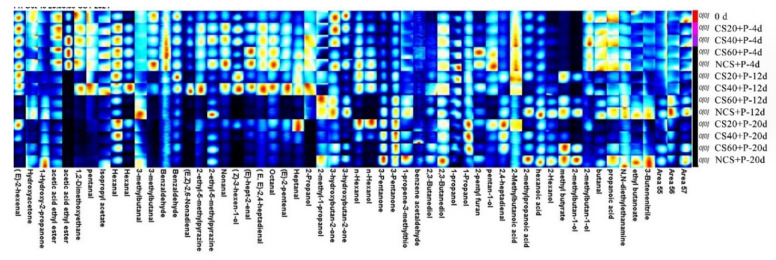
Fingerprints of volatile organic compounds in cucumbers under different treatments. The color depth represents the concentrations of the volatile compounds. Red indicates that the concentration of the substance is high, while blue indicates that the concentration of the substance is low. CS20+P−packaged after 20 min of cold shock; CS40+P−packaged after 40 min of cold shock; CS60+P−packaged after 60 min of cold shock; NCS+P−packaged after 0 min of cold shock.

As shown in Figure 8, each row of the figure indicates all the signals selected from the cucumber samples, and each column indicate the signal peak intensity of the same volatile component in cucumber samples with different storage times for different treatments. As can be seen in Figure 8, the volatile flavor substances in cucumbers of different treatments changed with the extension of storage time for each treatment. The contents of trans-2-hexenal, hydroxyacetone, ethyl acetate, ethylene glycol dimethyl ether, glutaraldehyde, isopropyl acetate, hexanal, 3-methylbutyraldehyde, benzaldehyde, and trans-cis-2,6-nonadienal showed different degrees of decrease. The aldehydes in the cucumber showed different degrees of decline with storage time, indicating a decrease in aroma significantly, probably due to microbial growth. These results indicated that the cucumber in each treatment showed different degrees of aroma loss during storage, while the aldehyde content of the cucumber in CS+P treatment groups decreased slowly compared to the NCS+P treatment, indicating that the cold stimulation combined with perforated spontaneous air conditioning packaging treatment helped the cucumber flavor retention during storage, where CS20+P and CS40+P treatments were better than the other two treatment groups. The concentrations of 2-ethyl-5-methylpyrazine, nonanal, cis-3-hexenol, (E)-2-heptenal, trans-2,4-heptadienal, octanal, trans-2-hexenal, heptanal, 2-propanol, isobutanol, 3-hydroxy-2-butanone, and n-hexanol first increased and then decreased. The reason for this change in the aldehydes of C6–C9 (the main aroma of cucumber) may be that cucumber had not yet reached full maturity at the beginning of storage, and its main aroma had not yet reached its peak, and then declined after reaching the peak perhaps due to microorganisms and the aging of the cucumber itself. The concentration of 3-pentanone, methallyl sulfide, phenylethanal, 2,3-butanediol, 1-propanol, 2-isobutyric acid, caprylic acid, 2-hexanol, methyl butyrate, 2-methyl-1-butanol, butyraldehyde, propionic acid, N,N-diethyl ethylamine, ethyl butyrate, and 3-butenenitrile in NCS+P group increased with storage time, which also may be as a result of microbial and self-metabolic influences. The high content of alcohols may be due to the reaction between acyl-CoA and alcohols, microbial action, and oxidative aging of fruits, catalyzed by ester alcohol-acyltransferase [49], as well as the high content of amines and acids with unpleasant odors, etc., indicating that the effect of CS+P treatment on flavor retention of cucumber was better than that of NCS+P treatment. In conclusion, CS combined with PM-PMAP treatment had a good effect on cucumber flavor retention, and CS20+P and CS40+P treatment groups had the best effect.

**Figure 9:**
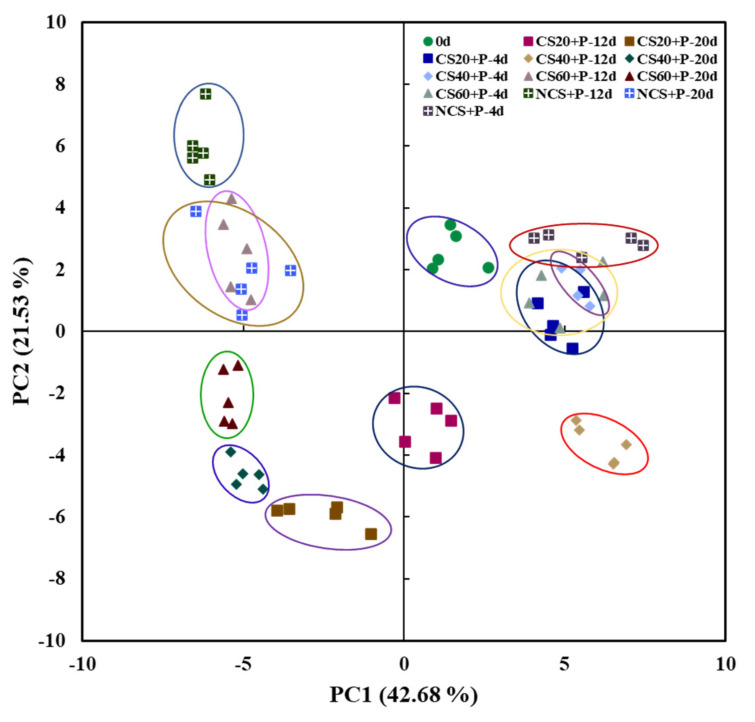
PCA scores of volatile organic compounds in cucumbers under different treatments. CS20+P−packaged after 20 min of cold shock; CS40+P−packaged after 40 min of cold shock; CS60+P−packaged after 60 min of cold shock; NCS+P−packaged after 0 min of cold shock.

PCA analysis is a method to reveal the intrinsic relationship between multivariate data or samples using the idea of dimensionality reduction to use a small number of integrated variables to replace the original complex multivariate quantities, to reduce data complexity [50], and to visualize the data. As shown in Figure 9, the contribution of the first principal component was 42.68%, and the contribution of the second principal component was 21.53%; the cumulative contribution of these two principal components was 64.21%, which represented most of the information of the original data. The relative proximity of the samples within the group indicated good reproducibility of the samples. The cucumbers of the four treatments were distributed in different areas, indicating that there were differences in VOCs among the samples, and the flavor of the cucumbers of the four treatments was changed more obviously with the extension of storage time. The flavor of cucumbers in all treatment groups on day 20 was more different compared with day 0. The flavor of cucumbers in the NCS+P treatment group changed most on day 20. The flavor of cucumbers in the CS20+P and CS40+P treatment groups at the end of storage was less different compared to day 0, indicating that CS20+P and CS40+P had a good effect on cucumber flavor retention.

**Figure 10:**
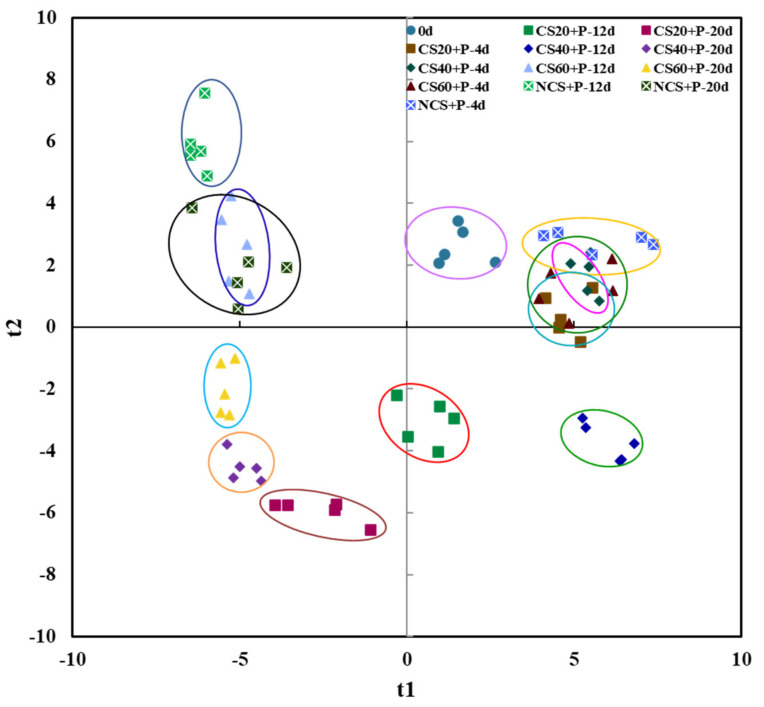
PLS–DA scores of volatile organic compounds in cucumbers under different treatments. CS20+P−packaged after 20 min of cold shock; CS40+P−packaged after 40 min of cold shock; CS60+P−packaged after 60 min of cold shock; NCS+P−packaged after 0 min of cold shock.

PLS–DA analysis is similar to PCA analysis in that it also performs a dimensionality reduction analysis method, but it can be pre-classified, which can remove the possible influence of uncontrolled variables on the data analysis [51], further mining the information in the data and quantifying the extent to which characteristic compounds cause component differences. As shown in Figure 10, after PLS–DA analysis, R^2^X was obtained as 0.976, R^2^Y as 0.851, and Q^2^ as 0.580, with the X matrix as the variable (VOCs) observation matrix, and the Y matrix as the category (cucumbers after different treatment) attribution matrix; here, R^2^X and R^2^Y indicate the percentages of X and Y matrix information explained by the model, respectively; Q^2^ represents the prediction rate of the model [52]. These indicated a model with a good stability and a good predictability for cucumbers with different treatments. It can be used for discriminant analysis of cucumber samples during storage.

Variable importance for the projection (VIP) can quantify the contribution of each variable of PLS–DA to classification. It is usually considered that VIP greater than 1 indicates an important role in the discriminatory process; and the larger the VIP value, the more significant the difference of variables among cucumbers of different treatments [53]. A panel of 20 VOCs with *p*-value < 0.05 and VIP score > 1 were selected as potential markers for the discrimination of cucumbers with different treatments. These 20 VOCs were 2-ethyl-5-methyl pyrazine, isobutyric acid, 2,3-butanediol, isobutanol, 1-propanol, benzaldehyde phenylacetaldehyde, 3-butenenitrile, 3-methylbutyraldehyde, 2-pentylfuran, 2-methyl-1-butanol, 2-hexanol, trans-2,4-heptadienal, trans, cis-2,6-nonadienal, 1-pentanol, hexanoic acid, ethyl acetate, ethyl butyrate, 3-pentanone, and methyl butyrate. The largest contributing VOC was 2-ethyl-5-methyl pyrazine, which was particularly important in distinguishing between different treatments of cucumber.

## 4. Conclusions

The data presented in this article show that the combination of CS and PM-PMAP treatment was beneficial to maintaining the quality of cucumber after harvest, such as minimal weight loss, color change, hardness, and total soluble solids reduction, as well as ascorbic acid retention, and malondialdehyde increment. Similarly, flavor change and respiratory rate were minimized. In summary, when the cold shock time was 40 min, the preservation quality of cucumber was the best. As a simple, safe, and inexpensive post-harvest processing technology, the use of CS combined with PM-PMAP to manipulate physiological and biochemical activities was found to be a practical method to extend the shelf life of cucumber fruits during the storage period. Future research may include different types of fruits and vegetables using the combined pretreatment method. The optimal processing parameters (cold shock intensity, time, and packaging materials) can be explored through comprehensive experiments.

## Figures and Tables

**Table 1:** Effect of different treatments on weight loss of cucumbers during storage at 13 ± 2 °C. CS20+P—packaged after 20 min of cold shock; CS40+P—packaged after 40 min of cold shock; CS60+P—packaged after 60 min of cold shock; NCS+P—packaged after 0 min of cold shock.

Storage Time (days)	Weight Loss (%)			
	CS20+P	CS40+P	CS60+P	NCS+P
0	0.00 ± 0.00 j	0.00 ± 0.00 j	0.00 ± 0.00 j	0.00 ± 0.00 j
4	0.14 ± 0.05 h,i	0.08 ± 0.01 i,j	0.15 ± 0.03 h,i	0.12 ± 0.08 h, i,j
8	0.20 ± 0.03 h,i	0.13 ± 0.02 h,i,j	0.25 ± 0.06 h	0.20 ± 0.03 h,i
12	0.74 ± 0.04 f	0.52 ± 0.06 g	0.62 ± 0.07 f,g	0.97 ± 0.14 d,e
16	1.00 ± 0.03 d,e	0.67 ± 0.06 f	0.94 ± 0.09 e	1.51 ± 0.04 c
20	1.90 ± 0.14 b	1.09 ± 0.06 d	1.93 ± 0.06 b	2.32 ± 0.27 a

Values with different letters in each row are significantly different (*p* < 0.05).

**Table 2:** Qualitative information of volatile organic compounds in cucumbers under different treatments CS20+P; CS40+P; CS60+P; and NCS+P. CS20+P−packaged after 20 min of cold shock; CS40+P−packaged after 40 min of cold shock; CS60+P−packaged after 60 min of cold shock; NCS+P−packaged after 0 min of cold shock.

Count	Compounds	Formula	MW	RI	Rt/s	Dt/ms
1	(E,Z)-2,6-nonadienal	C_9_H_14_O	138.2	1160.1	841.643	1.37517
2	nonanal	C_9_H_18_O	142.2	1106	722.887	1.47299
3	(E, E)-2,4-heptadienal	C_7_H_10_O	110.2	1017.3	528.284	1.19128
4	2-ethyl-5-methylpyrazine	C_7_H_10_N_2_	122.2	1004.3	499.76	1.19805
5	2,4-heptadienal	C_7_H_10_O	110.2	990.3	473.833	1.18517
6	hexanoic acid	C_6_H_12_O_2_	116.2	989.7	472.939	1.30197
7	benzaldehyde	C_7_H_6_O	106.1	967.1	440.753	1.14867
8	octanal	C_8_H_16_O	128.2	1005.4	502.228	1.39972
9	2-ethyl-5-methylpyrazine	C_7_H_10_N_2_	122.2	1005.4	502.352	1.67368
10	(E)-hept-2-enal	C_7_H_12_O	112.2	956	424.963	1.25224
11	(E)-2-hexenal	C_6_H_10_O	98.1	851.2	296.851	1.17821
12	(Z)-3-hexen-1-ol	C_6_H_12_O	100.2	847.5	293.517	1.51382
13	n-hexanol	C_6_H_14_O	102.2	875.5	318.735	1.32264
14	hexanal	C_6_H_12_O	100.2	793.2	244.5	1.25877
15	2-methylpropanoic acid	C_4_H_8_O_2_	88.1	772.1	228.908	1.15595
16	(E)-2-pentenal	C_5_H_8_O	84.1	746.8	213.315	1.10294
17	3-hydroxybutan-2-one	C_4_H_8_O_2_	88.1	719.7	196.612	1.06159
18	3-hydroxybutan-2-one	C_4_H_8_O_2_	88.1	717.1	194.999	1.32669
19	3-pentanone	C_5_H_10_O	86.1	688.9	177.662	1.11033
20	3-pentanone	C_5_H_10_O	86.1	687.2	176.856	1.35328
21	3-methylbutanal	C_5_H_10_O	86.1	642.7	158.107	1.1982
22	1,2-dimethoxyethane	C_4_H_10_O_2_	90.1	646.7	159.822	1.29793
23	acetic acid ethyl ester	C_4_H_8_O_2_	88.1	608.5	143.747	1.09566
24	acetic acid ethyl ester	C_4_H_8_O_2_	88.1	605.4	142.442	1.33452
25	1-propanol	C_3_H_8_O	60.1	570.2	127.59	1.11288
26	butanal	C_4_H_8_O	72.1	592.3	136.896	1.28754
27	2,3-butanediol	C_4_H_10_O_2_	90.1	792.5	243.869	1.36382
28	3-methylbutanal	C_5_H_10_O	86.1	645.7	159.384	1.39846
29	n-Hexanol	C_6_H_14_O	102.2	874.5	317.831	1.63758
30	hexanal	C_6_H_12_O	100.2	792.5	243.89	1.55909
31	1-propene-3-methylthio	C_4_H_8_S	88.2	700.9	185.033	1.04133
32	2-hexanol	C_6_H_14_O	102.2	810.1	259.734	1.58033
33	pentan-1-ol	C_5_H_12_O	88.1	766.5	225.463	1.24878
34	2-methyl-1-propanol	C_4_H_10_O	74.1	625.4	150.847	1.17441
35	benzaldehyde	C_7_H_6_O	106.1	969	443.513	1.46407
36	2-methylbutanoic acid	C_5_H_10_O_2_	102.1	876.5	319.684	1.47052
37	heptanal	C_7_H_14_O	114.2	901	346.449	1.32688
38	benzene acetaldehyde	C_8_H_8_O	120.2	1054.9	610.944	1.25142
39	2-pentyl furan	C_9_H_14_O	138.2	992.9	477.61	1.25149
40	N,N-diethylethanamine	C_6_H_15_N	101.2	688.7	177.505	1.21985
41	2-methylbutan-1-ol	C_5_H_12_O	88.1	739.1	208.58	1.46753
42	2,3-butanediol	C_4_H_10_O_2_	90.1	826.6	274.661	1.36211
43	methyl butyrate	C_5_H_10_O_2_	102.1	741.9	210.305	1.15562
44	ethyl butanoate	C_6_H_12_O_2_	116.2	759.3	221.019	1.20389
45	3-butenenitrile	C_4_H_5_N	67.1	621.5	149.205	1.12862
46	propanoic acid	C_3_H_6_O_2_	74.1	742.2	210.487	1.27215
47	hydroxyacetone	C_3_H_6_O_2_	74.1	718.6	195.924	1.22934
48	2-propanol	C_3_H_8_O	60.1	537.8	113.95	1.16127
49	1-hydroxy-2-propanone	C_3_H_6_O_2_	74.1	671	170.026	1.04279
50	1-propanol	C_3_H_8_O	60.1	562.7	124.462	1.25704
51	2-methylbutan-1-ol	C_5_H_12_O	88.1	736	206.685	1.24314
52	Isopropyl acetate	C_5_H_10_O_2_	102.1	657.1	164.2	1.16453
53	pentanal	C_5_H_10_O	86.1	690.5	178.596	1.18696

## Data Availability

The data presented in this study are available in article.
